# Ferroelectric thin film acoustic devices with electrical multiband switching ability

**DOI:** 10.1038/s41598-017-14895-8

**Published:** 2017-11-10

**Authors:** Sergey V. Ptashnik, Anatoliy K. Mikhailov, Alexander V. Yastrebov, Peter K. Petrov, Wei Liu, Neil McN Alford, Soeren Hirsch, Andrey B. Kozyrev

**Affiliations:** 1 Microwave laboratory “Pulse”, Saint-Petersburg state electrotechnical university, Saint-, Petersburg, Russia; 2 Dagestan University of National Economy, Makhachkala, 367008 Russia; 3 Department of Materials, Imperial College, London, United Kingdom; 4 Department of Engineering, University of Applied Sciences Brandenburg, Brandenburg, Germany

## Abstract

Design principles of a new class of microwave thin film bulk acoustic resonators with multiband resonance frequency switching ability are presented. The theory of the excitation of acoustic eigenmodes in multilayer ferroelectric structures is considered, and the principle of selectivity for resonator with an arbitrary number of ferroelectric layers is formulated. A so called “criterion function” is suggested that allows to determine the conditions for effective excitation at one selected resonance mode with suppression of other modes. The proposed theoretical approach is verifiedusing thepreexisting experimental data published elsewhere. Finally, the possible application of the two ferroelectric layers structures for switchable microwave overtone resonators, binary and quadrature phase-shift keying modulators are discussed. These devices could play a pivotal role in the miniaturization of microwave front-end antenna circuits.

## Introduction

Thin film ferroelectric bulk acoustic resonators (FBAR) offer a significant potential in modern microwave electronics. The key features of the FBARs are their ultra-small size and the ability to work at frequencies up to tens of gigahertz. They are widely used in miniaturized filters for communication and navigation systems. Modern microwave FBARs are multilayer thin film structures containing one piezoelectric layer (AlN, ZnO)^1–3^. The operating frequency of these resonators is determined by the thickness of the structure and by the elastic properties of the layers, and cannot be electrically switched or effectively tuned. Therefore, the development of FBARs with capability of electrical control of their resonance frequency is a challenge whose solution could lead to a significant improvement of modern filter devices and microwave systems^4^.

A possible solution of this problem is to replace the piezoelectric materials that are traditionally used for FBAR fabrication with ferroelectric materials in the paraelectric phase. A good candidate is barium-strontium titanate (Ba_x_Sr_1−x_TiO_3_, BSTO)^4–11^, Due to its electric field induced piezoelectricity with relatively high values of electromechanical coupling coefficient that allows for the design of tunable and switchable FBARs. Furthermore, by varying the Barium to Strontium stoichiometric ratio, one could alter the BSTO material properties and enhancing eitherthe piezoelectric coefficient (by increasing the Barium content) or the electrical/acoustical quality factor (by increasing the Strontium content).

**Figure 1:**
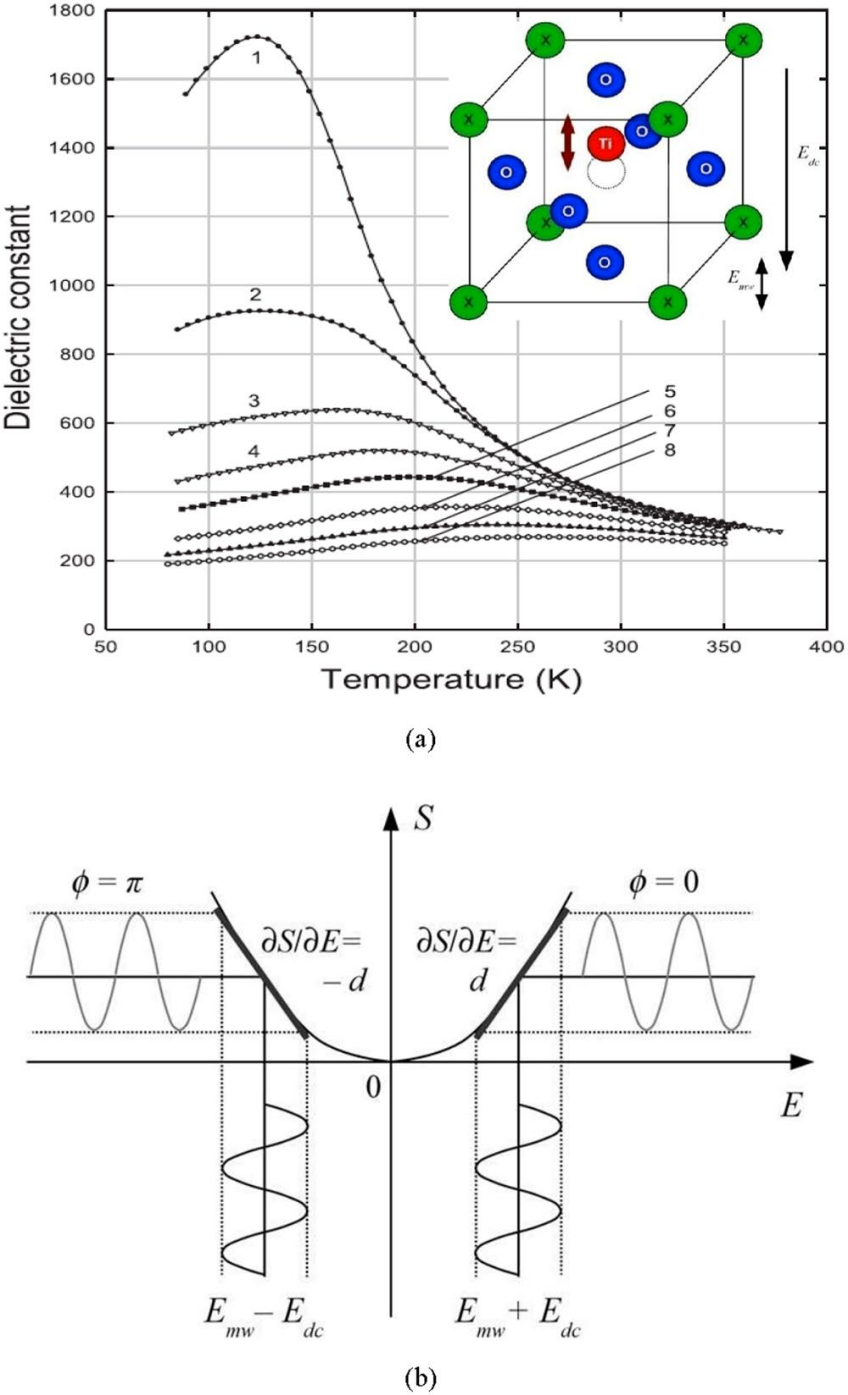
(**a**) Dielectric constant of the Ba_0.3_Sr_0.7_TiO_3_ film as a function of temperature under electric fields (experimental data), E (V/µm): (1)—0, (2)—5, (3)—10, (4)—20, (5)—30, (6)—40, (7)—50, and (8)—60. The crystal cell of Ba_x_Sr_1−x_TiO_3_ (X is Ba or Sr) and shift of the titan atom under bias field (E_dc_) and it oscillations under microwave field (E_mw_) are shown in the insert. (**b**) Dependence of strain vs electric field. Change of E_dc_ polarity results in 180° change of phase of microwave signal.

The BSTO has a perovskite crystal structure with Ti atom in the center of lattice cell (see insert in Fig. 1(a)). In the paraelectric phase (i.e. at temperatures above T_c_, where T_c_ is Curie temperature) and in the absence of an applied electric field, Ti is in the central position, the system is symmetrical, and BSTO does not have piezoelectric properties. In the ferroelectric phase or under external electric field, Ti shifts from the central position, which leads to the loss of symmetry and the appearance of the piezo-electric effect. In the case when the shift is caused by external electric field, the piezoelectric effect is called an induced piezo-effect. Anundesirable feature of the ferroelectricmaterials is the strong temperature dependence of permittivity ε(T) near the temperature of their ferroelectric-paraelectric phase-transition (Fig. 1(a)). However, in BSTO films, with compositions x = (0.1–0.5), under dc electric fields E_dc_ ≥ 40 V/μm (corresponding to the maximum values ofthe electromechanical coupling coefficient),this undesired effect is suppressed; Fig. 1a. The phenomenon of induced piezo-effect (also known as “linearized electrostriction”) is illustrated in Fig. 1(b). In this figure, the parabolic electrical field (E) dependence of the mechanical strain (S) corresponding to electrostriction effect is shown. Let us assume that electric field is a sum of two components E = E_dc_ + E_mw_, where E_dc_ – constant bias, E_mw_ – microwave (MW) signal. At |E_mw_| ≪ |E_dc_|, the S microwave response can be approximated as linear, and the value and the sign of the derivative *∂S/∂E* define the piezo-electric coefficient (d) at different E_dc_ operating points. Beside the d variations under E_dc_, the other field-dependent material parameters, such as permittivity (ε) and stiffness (c), are changed under E_dc_ that results in the tuning of the resonance frequency of BSTO FBAR^5–11^.

**Figure 2:**
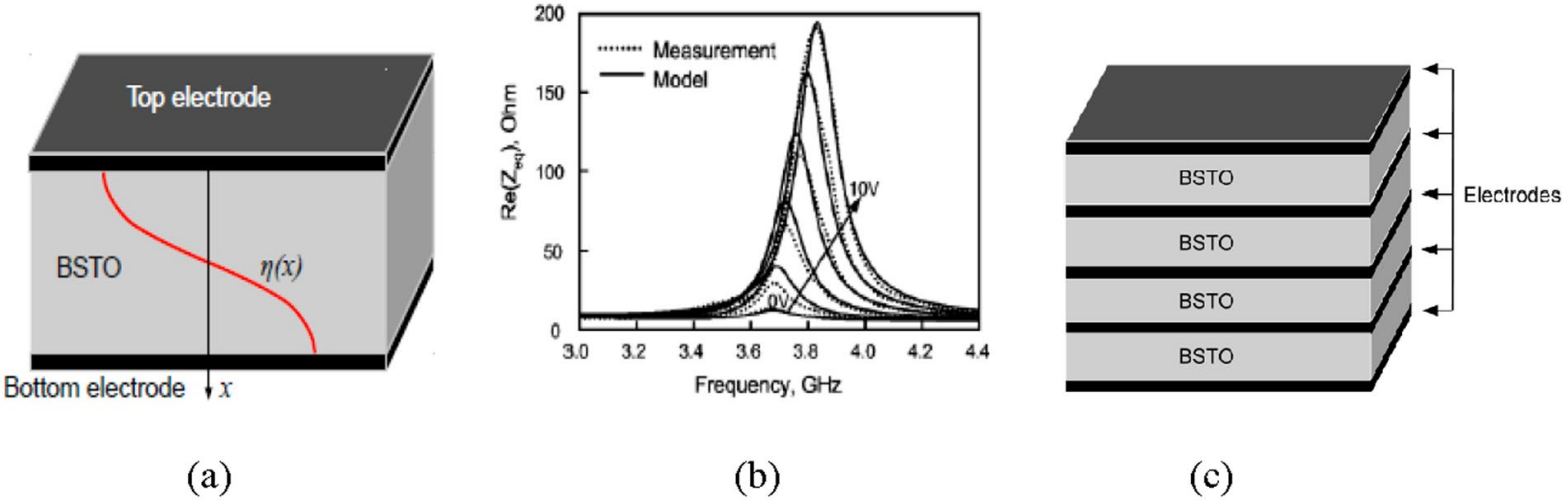
Schematic of film bulk acoustic wave resonator with one BSTO layer, and displacement profile (η(*x*)) for first normal acoustic mode (**a**). Frequency dependence of the impedance of the BSTO FBAR at different bias field voltages^7^ (**b**), (reproduced from I. B. Vendik, P. A. Turalchuk, and O. G. Vendik, Journal of Applied Physics 103, 014107 (2008) with the permission of AIP Publishing); multilayer thin film FE structure with conductive electrodes between layers (**c**).

Both theoretical and experimental results predict a maximum electrical tuning up to 5% of the FBAR’s operating frequency by varying the magnitude of the applied bias field^5–10^. A typical graph illustrating the frequency tuning of a one BSTO layer resonator (Fig. 2a) under bias voltage is shown in Fig. 2(b). However, in practical applications, tuning the resonant frequency by 5% is problematic due to the significant variation of the input impedance of the FBAR that inevitably leads to a mismatch of the microwave circuit. However, for a set of applications the use of this phenomena for small (up to (1–2)%) frequency tuning is desirable^9^.

A promisingtuning method was developed by group of prof A.Mortazawi^12^. They proposed to use multiple filters based on “on/off switchable” BSTO FBARs each of those designed to work on a specific frequency from the required set of frequencies. These filters are linked in a parallel circuit (filter bank) and, in each moment of time, DC bias is applied to the resonators of only one filter, while other filters are in “off” state. In this case, the frequency band of the entire circuit is defined by the band of the “on-switched” filter. This approach do not suffer from impedance mismatchingproblem noted above.

In this paper, another approach based on the use of induced piezoelectric phenomena in ferroelectrics is considered. The main idea is the use of multilayer thin film FE structure with conductive electrodes between layers, which make possible to apply E_dc_ of different polarities and magnitudes to each FE layer or groups of FE layers (Fig. 2(c)). This results in the ability to change the sign and value of its piezo-coefficients (Fig. 1(b)) and allows enormous switching of the FBAR’s operation frequency.

Despite the number of publications on the induced piezo-effect in ferroelectrics^5–11^, the theoretical description (mathematical model) of the behaviour of multilayer ferroelectric film structures with a possibility of resonance frequency switching has not been sufficiently developed. There is a number of models based on simple equivalent circuits, such as Butterworth-Van Dyke (BVD and mBVD models)^3^. However they consider single layer structures in a limited frequency range. Of course, there are more advanced models (e.g. refs^13,14^), which use becomes over complicated when applied to structure with more than one piezoelectric layer, and especially if the structure employs tunable ferroelectric layers. Therefore, thefirst part of this paper presents a mathematical description of FBAR structures with two- and arbitrary number (*n*) of FE layers.

One of the main requirements for frequency switchable resonators is the presence of excitations at one selected resonance mode and suppression of other modes. So, further in the paper, the principle of selectivity for a resonator with an arbitrary number (n-layer) of FE active layers is formulated. A so-called “criterion function” is proposed, which makes possible to determine the magnitudes and polarities of the control voltages applied to each FE layer to provide the excitation or suppression of operating resonance modes. As an example, the expected multiband switching ability of a FBAR with four active FE layers is considered. We validated the proposed modelby comparisonof the results of our calculationwith the data from previousexperimental works^15–17^. Finally, the possible applications of two layer structures for switchable MW high overtone bulk acoustic resonator (HBAR), binary and quadrature phase-shift keying (BPSK, QPSK) modulators are suggested.

## Theory

The possibility to provide the frequency switching of two FE layer BAR due to the application of the control voltages of different polarities to layers has been earlier experimentally demonstrated for RF^16^ and MW^15^ frequency ranges. The mathematical approach considered below models the acoustic MW response of thin film structures with an arbitrary number of ferroelectric layers with induced piezoelectric effect when electric fields E_dc_ with different magnitudes and polarities are applied to each FE layer or to a group of FE layers.

The model is based on equations that describe relations between electrical and mechanical processes in a piezoelectric^3^:

1 $$\begin{array}{rcl}T & = & {c}^{E}S-eE\\ D & = & eS+{\varepsilon }^{S}E\end{array}$$

where T is the stress, S is the mechanical strain, E is the electric field intensity, D is the electric displacement field, c^E^ is the mechanical stiffness at the constant electric field, *e* = −*(∂T/∂E)* ^*S*^ is the piezoelectric coefficient (linked with piezoelectric coefficient *d* = *(∂S/∂E)* ^*T*^ as *e* = *dc* ^*E*^), ε^S^ is the clamped permittivity (below referenced simply as ε). Note that in 3D case T, S, E and D will be vector fields, and c^E^, e and ε^S^ will be tensors. However, for a thin film structure, where the thickness is much less than the lateral dimensions, the 1D approach is acceptable (*x* axis is orthogonal to the plane of the film). In that case, all these parameters will be scalars.

The set of mathematical transformations of (1) results in the system of differentialequations^1^:

2 $$\begin{array}{rcl}\frac{\partial T}{\partial x} & = & {c}^{E}\frac{{\partial }^{2}\eta }{\partial {x}^{2}}+e\frac{{\partial }^{2}\phi }{\partial {x}^{2}}=\rho \frac{{\partial }^{2}\eta }{\partial {t}^{2}}\\ \frac{\partial D}{\partial x} & = & e\frac{{\partial }^{2}\eta }{\partial {x}^{2}}-\varepsilon \frac{{\partial }^{2}\phi }{\partial {x}^{2}}=0\end{array}$$

where *η*(*x*) is the mechanical displacement in point with coordinate *x* (note that it linked with strain by $S=\partial \eta /\partial x$). For harmonic processes, the time dependencies of *η* and *φ* can be expressed via multiplication by *e* ^*jωt*^, where ω is the circular frequency of oscillations.

The solution of (2) is following:

3 $$\begin{array}{rcl}\eta (x) & = & {\beta }_{-}{e}^{jkx}+{\beta }_{+}{e}^{-jkx}\\ \phi (x) & = & \frac{e}{\varepsilon }\eta (x)\,-\,\alpha x+C\\ T(x) & = & j\omega {Z}_{a}({\beta }_{-}{e}^{jkx}-{\beta }_{+}{e}^{-jkx})-e\alpha \end{array}$$

where β_+_ and β_−_ are the amplitudes of incident and reflected waves respectively, $k=\omega /V$ is the wave number ($V=\sqrt{{c}^{D}/\rho }$ is the speed of sound in material, ${c}^{D}={c}^{E}+{e}^{2}/\varepsilon$ is the material stiffness at the constant electric displacement), ${Z}_{a}=\rho V$ is the acoustic impedance, α and C are constants of integration. α corresponds to coordinate-independent component of electric field strength as in a simple dielectric capacitor.

The structure can contain any number (n) of layers. In any of these regions, the electromechanical processes will be described by (3). Each region will have its own values of material parameters (ρ_i_, c_i_^E^, ε_i_, e_i_) and, hence, its own value of *k*_i_.

To determine unknown values of (3), that are β_*i*+_, β_*i*−_ and α_*i*_, the boundary conditions must be formulated considering that mechanical displacements η and mechanical stress T are continuous across the layer interfaces:

4 $$\begin{array}{rcl}{\eta }_{i}({x}_{i}) & = & {\eta }_{i+1}({x}_{i})\\ {T}_{i}({x}_{i}) & = & {T}_{i+1}({x}_{i})\\ i & = & 0..\,n\end{array}$$

where, *x*_*i*_ is the coordinate of boundary between *i*-th and (*i* + 1)-th layers (*i* = 0 and *i* = *n* + 1 correspond the boundaries with external medium).

To obtain a fully determined system, it is necessary to add a corresponding number of conditions to define values of α_*I*_ in the system. This can be done due to potential differences of all dielectric layers:

5 $${U}_{i}={\phi }_{i}({x}_{i+1})\,-\,{\phi }_{i}({x}_{i})$$

where, U_*i*_ is amplitude of MW signal, applied to *i*-th layer. Having the full system of boundary conditions (4) and (5), we can obtain values of β_*i*−_, β_i+_ and α_*i*_ of each dielectric layers. Then, we can use these values to determine the electrical impedance of the dielectric layers

6 $${Z}_{i}=\frac{{U}_{i}}{{I}_{i}}=\frac{{\phi }_{i}({x}_{i+1})\,-\,{\phi }_{i}({x}_{i})}{j\omega {\alpha }_{i}{C}_{i}{h}_{i}}$$

where, *h*_*i*_ is the thickness of *i*-th layer, ${C}_{i}=\varepsilon A/{h}_{i}$ is their capacitance (A is the area of resonator).

The influence of E_dc_ on the piezocoefficient is

7 $$\begin{array}{rcl}e({P}_{dc}) & = & 2G\varepsilon ({P}_{dc}){P}_{dc}\\ \varepsilon ({P}_{dc}) & = & \frac{1}{1/\varepsilon (0)+3\beta {P}_{dc}^{2}}\end{array}$$

where, *P*_*dc*_ = χ_*e*_ *E*_*dc*_ is the polarization induced by *E*_*dc*_ (χ_*e*_ is the electric susceptibility), *ε*(0) is the electric permittivity at zero field, G is the electrostriction coefficient, *β* is a coefficient of nonlinearity of the ferroelectric. The relations in (7) are only rough approximations of the dependencies used in refs^5–10^ for the description of frequency tuning of one FE layer resonators and does not consider high-order components. However, it is sufficient for consideration of the switching phenomena in a multilayer FE structure.

The specific feature of our approach is the use of an artificial concept, namely the variation of the sign of the piezoelectric coefficient in FE layers. This allows to consider the influence of *E*_*dc*_ polarity on the phase-shift between electrical and acoustic oscillations. As one can see from Fig. 1(b), the switching of *E*_*dc*_ polarity from positive to negative leads to 180° phase shift in acoustic response. It can be mathematically described by a negative value of the piezoelectric coefficient. As it will be shown below, this concept allows us to describe correctly the phenomenon of switching the resonant frequency in a structure that has two and more FE layers with induced piezoelectricity.

## Multilayer structures

**Figure 3:**
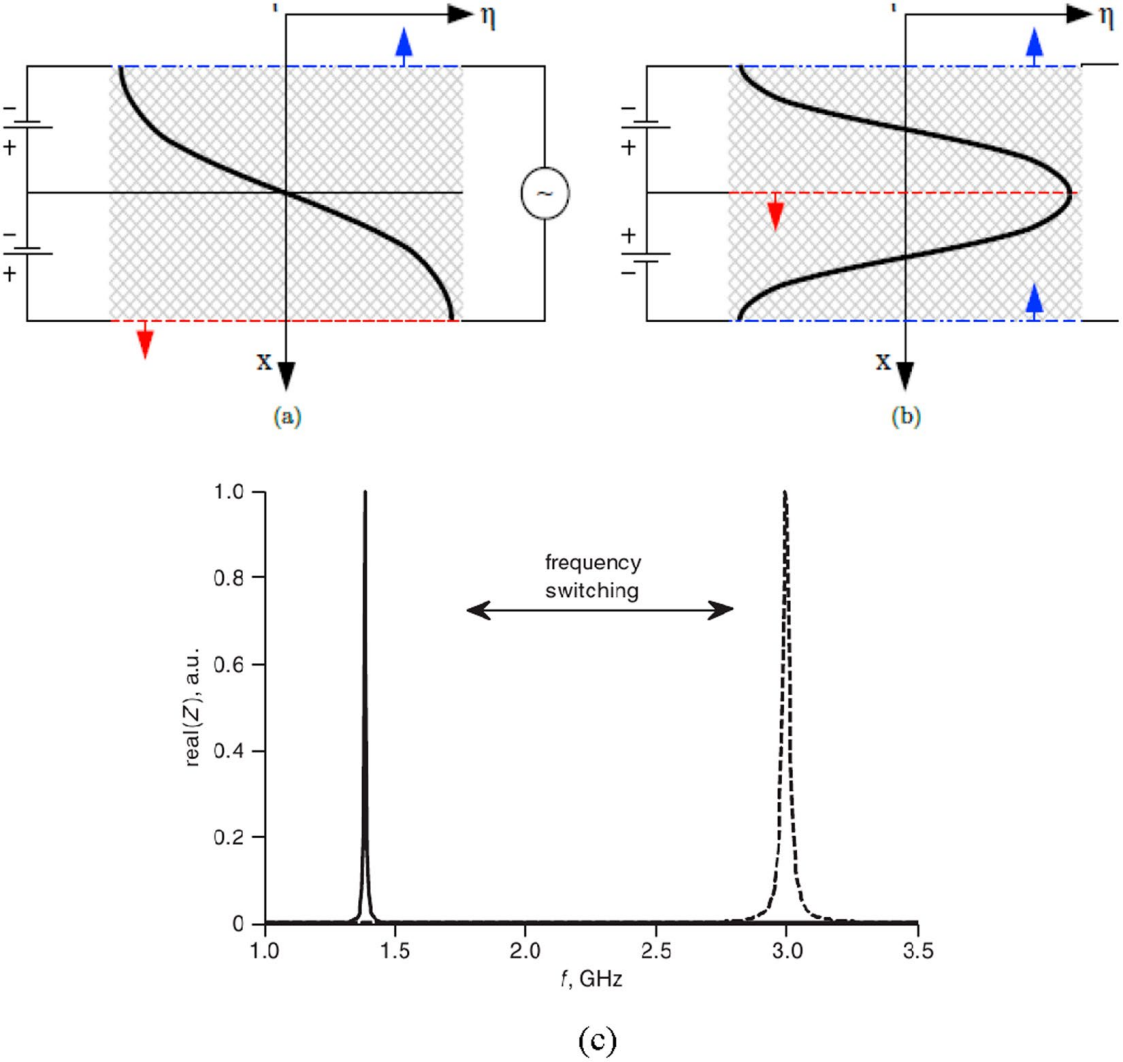
Excitation of odd (**a**) and even (**b**) normal modes in two layer FBAR. Arrows demonstrate the boundary movement. Frequency dependences of the real part of electric impedance of the model SrTiO_3_ (180 μm Pt/0.8 μm STO/0.05 μm Pt/0.8 μm STO/0.2 μm Pt) resonator at different mutual bias polarities illustrating frequency switching phenomena (**c**), (reproduced from A. Kozyrev, A. Mikhaylov, P. Petrov, and N. Alford, Electronics Letters 47, 1326 (2011), with the permission of IET).

Let us consider the two FE layer structure (Fig. 3). At the same base polarity of layers (the same sign of the piezo-coefficient) the resonator operates like traditional one layer FE device, i.e. only odd normal acoustic modes will be excited (1^st^, 3^rd^, etc.) (Fig. 3a). At the base voltages of opposite polarity (different sign of *d* in layers), the antiphase oscillation of layers must be observed, so the odd modes of each layer will correspond to even modes (2^nd^, 4^th^ etc.) of the entire structure (Fig. 3b). Therefore, the switching of control polarity of layers will result in switching between two frequency spectra of excitation (Fig. 3c). In resonators with two FE layers, the selective excitation of 1^st^ and 2^nd^normal modes is relatively simple and can be achieved by changing the polarity of the control voltage. For operation at more than two resonance frequencies, we need to use a more advanced FE multilayer structure (e.g. Fig. 2c), where one has to control not only the polarity of the DC voltage applied to each layer, but also the magnitude of the voltage applied to each layer or groups of layers.

**Figure 4:**
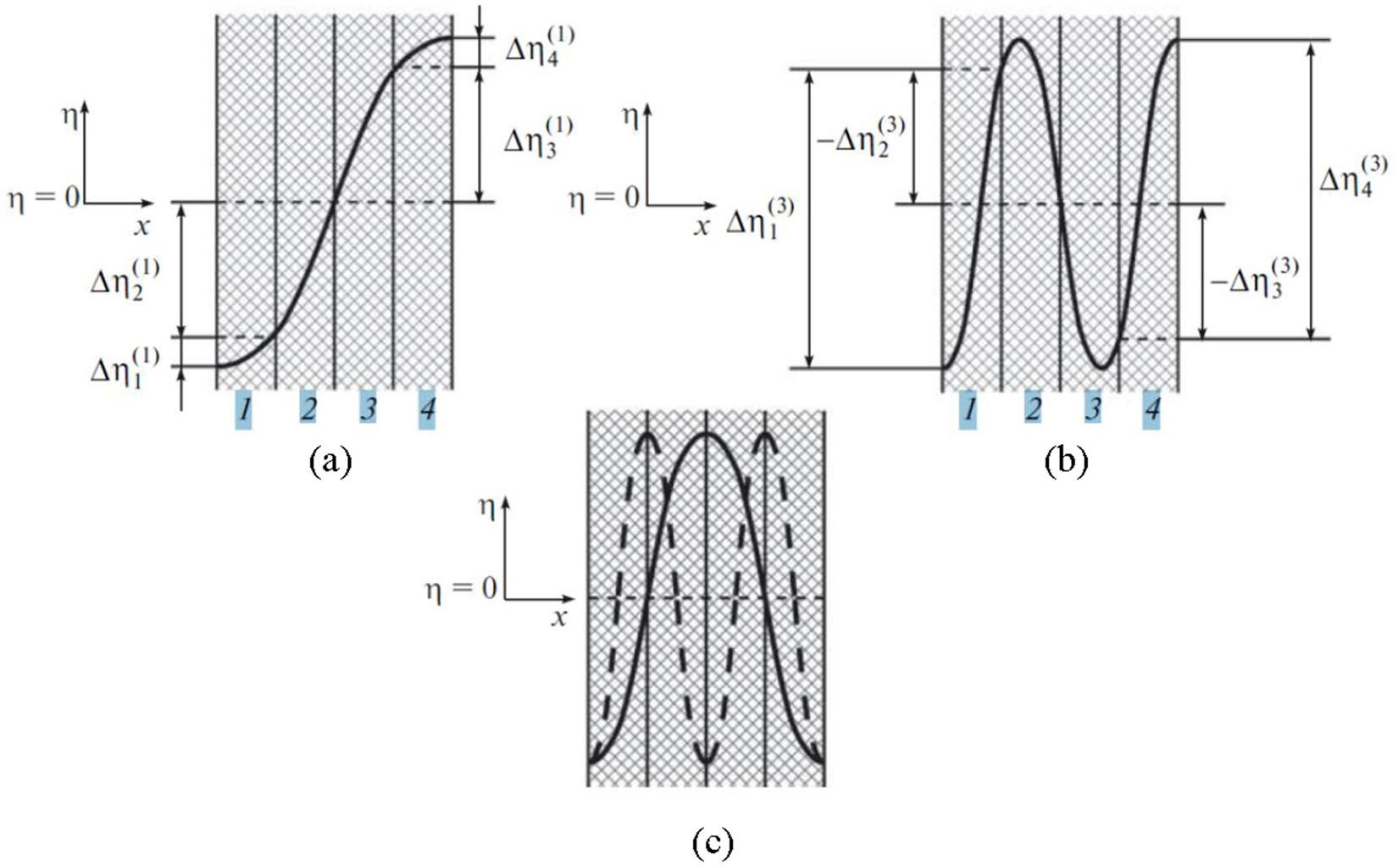
Examples of determining ${\rm{\Delta }}{\eta }_{j}^{(i)}$ for (**a**) first and (**b**) third acoustic eigenmodes, as well as (**c**) second and fourth eigenmodes in a four-layer BAW resonator structure. (reproduced from A. Mikhaylov *et al*., Technical Physics Letters, 42, 8 (2016), with the permission of TPL).

**Figure 5:**
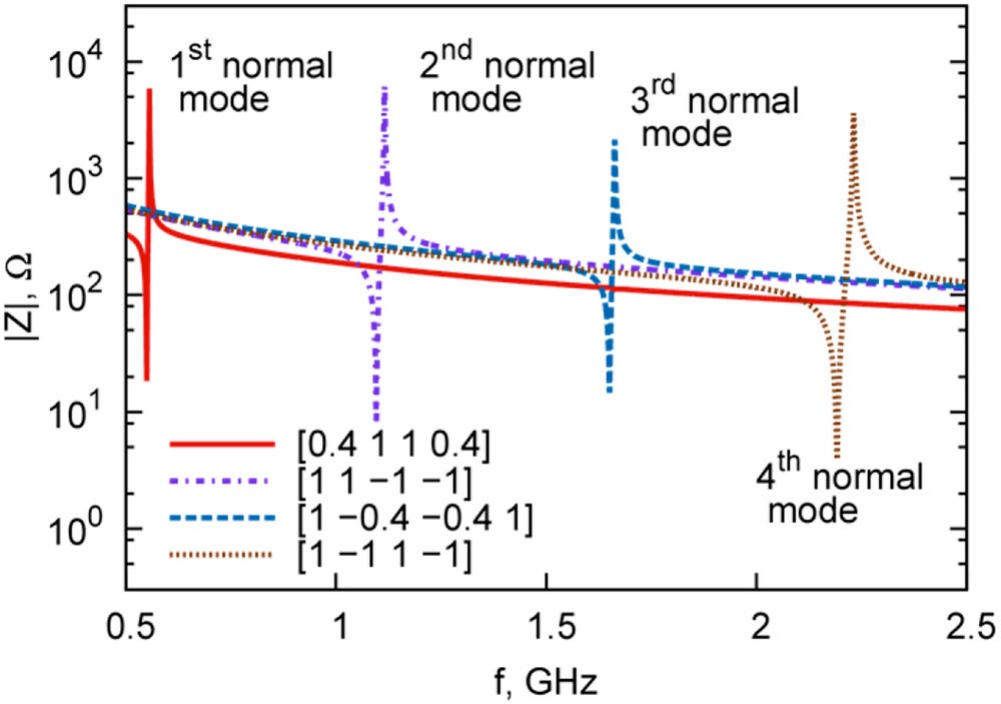
Frequency dependences of the real part of electric impedance of the model four-layer SrTiO_3_ resonator (four 1 μm STO layers) for different combinations of the polarities and absolute values of bias fields applied to each layer (values of bias fields are normalized to 40 V/μm) (reproduced from A. Kozyrev, A. Mikhailov, S. Ptashnik, P. K. Petrov, and N. Alford, Applied Physics Letters 105, 162910 (2014), with the permission of AIP Publishing).

Multilayer (n-layer) FE structures allow the application of control voltages to each layer or groups of layers and result in an expansion in functionality of FBAR resonators. For example, let us consider the principle of control over a four-layer structure (*n* = 4). The schematic diagram of this structure and the scheme of application of bias voltages and microwave signal are presented in Fig. 4. When bias fields of the same sign are applied to all layers, the structure operates as a single oscillator with excited mode numbers 1 (Fig. 4a), 3 (Fig. 4b), 5, etc. If bias fields of the same sign are applied to the first two layers, and fields of the opposite polarity are applied to the other two layers, the structure operates as a system of two mechanically coupled resonators with excited mode numbers 2 (Fig. 4c, solid curve), 6,10, etc. When bias fields of alternating signs are applied to all sequential layers, the structure operates as a system of four resonators with excited mode numbers 4 (Fig. 4c, dashed curve), 12, 20, etc.

The efficiency of mode excitation is defined by the maximum of the amplitude of the standing acoustic wave and the absolute peak value of the electric impedance (or admittance) of the resonator. The higher these values are, the more effective is the excitation of the selected mode. A comparison of the peak values of impedances for the different resonance modes allows to formulate the principle of efficient selectivity: the most effective excitation of the selected n-the eigenmode in a resonator comprising *n* piezo-active layers with identical acoustic wave phase incursion and mechanical impedance is achieved when alternating polarity of control voltages is applied to sequential layers. So, the most effective excitation of 2^nd^, 3^rd^, 4^th^ etc. eigenmodes will correspond to 2, 3, 4 etc. FE layer structures, respectively.

For practical applications, it is of interest to create a resonator switchable between several regimes (within an operating frequency range) with a single mode excited in each one. One of the main requirements for frequency switchable resonators is excitations at one selected resonance mode and suppression of other modes. For a resonator with four ferroelectric layers, this task is successfully solved for the second and fourth modes by selecting the appropriate bias field polarities in accordance with the principle of selectivity that is formulated above. However, the “separation” of the first and third modes (Fig. 4a and b), i.e. creation of conditions under which either the first mode is excited and the third mode is suppressed or vice versa, does not conform to this principle of selectivity.The problem of “separation” of the first and third modes is a particular case of the general problem of selective excitation of a given mode in a resonator structure with an arbitrary number of layers with suppression of several “adjacent” modes that are close in frequency. To solve this general problem, we propose an approach based on the search for extremes of the following functional:

8 $${f}^{i}(c(x))=|{\int }_{0}^{h}{\rm{\Delta }}{\eta }^{(i)}(x)c(x)dx|$$

where variable *c*(*x*) determines the effect of electric field on the medium (normalized piezoelectric coefficient) at point *x* and $\partial {\eta }^{(i)}(x)/\partial x$ represents the response–i.e., the spatial distribution of the amplitude of mechanical deformation for the i^th^ mode (derivative of the mechanical displacement $\partial {\eta }^{(i)}(x)$ with respect to coordinate for the i^th^ mode). The integration limits (0, *h*) in this expression correspond to coordinates of the structure boundaries on the *x* axis.

While $\partial {\eta }^{(i)}(x)/\partial x$ determined by the selected mode that should be excited or suppressed, the term *c*(*x*) is a tool that is capable to provide effective excitation $({f}^{i}(c)\to \,\max )$ or suppression $({f}^{i}(c)\to \,\min )$ of the selected mode. Since the function c(x) is restricted to a stepwise-constant profile (with constant regions corresponding to layers and possible discontinuities at the interfaces between layers), it can be simplified as follows:

9 $${f}^{i}(\overrightarrow{c})=|\sum _{j=1}^{n}{\rm{\Delta }}{\eta }_{j}^{(i)}{c}_{j}|$$

where ${\rm{\Delta }}{\eta }_{j}^{(i)}$ is the amplitude of mutual oscillations of j^th^ layer boundaries on the i^th^ mode (Fig. 4a,b) with the sign that takes into account the mutual phases of oscillations in adjacent layers, ${\rm{\Delta }}{\eta }_{j}^{(i)}{\rm{\Delta }}{\eta }_{j+1}^{(i)} > 0$ if the j^th^ and (*j* + 1)^th^ layers oscillate in phase, and <0 if they oscillate in counter phase and

10 $${c}_{j}=\pm \frac{{e}_{j}({E}_{dc})}{{e}_{{\rm{\max }}}}$$

is normalized piezoelectric coefficients in the layers. Here, *E*_*dcj*_ is the electric bias field applied to the j^th^ layer, e_max_ is the maximum piezo-coefficient possible in the given structure, and the sign is determined by the polarity of the applied bias field.

Upon calculation of the spatial distributions of mechanical displacements ${\eta }^{(i)}(x)$ corresponding to acoustic eigenmodes of the given structure and determination of ${\rm{\Delta }}{\eta }_{j}^{(i)}$ for the piezoactive layers, it is possible to find the values corresponding to maxima of function (9) for the selected mode – that is, to the most effective excitation of this mode. Thus, expression (9) can be used as the criterion function for selecting optimum *c*_*j*_ (and, hence, *E*_*dcj*_) values corresponding to effective excitation of some eigenmodes and suppression of other modes.

For the four-layer resonator considered above, it is necessary to solve two optimization problems: maximization of the criterion function for a selected (first or third) eigenmode and provision of its minimization for the competitive (third or first, respectively) mode (minimax problem). Solution of these problems provides the values of control voltages on all layers of the resonator structure. In the framework of the above example, we have solved the following problems:

11 $$\begin{array}{c}\begin{array}{ll}\{\begin{array}{l}{f}^{1}(c)\to \,\max \\ {f}^{3}(c)=0\\ -1\le {c}_{j}\le 1\end{array} & \{\begin{array}{l}{f}^{3}(c)\to \,\max \\ {f}^{1}(c)=0\\ -1\le {c}_{j}\le 1\end{array}\end{array}\\ j=1..\,4\end{array}$$

which describe the most effective excitation of the first eigenmode at complete suppression of the third mode and the most effective excitation of the third eigenmode at complete suppression of the first mode. Solutions of these problems are represented by vectors *c* ≈ [0. 1 1 0.]^T^ and *c* ≈ [1–0.4–0.4 1]^T^, respectively.

Figure 5 shows the results of modeling of the frequency dependences of the real part of electric impedance of the given resonator for four combinations of the polarities and absolute values of bias fields, optimized using criterion (9) for excitation of the four lower modes of the resonator. As can be seen from these data, the model structure comprising four SrTiO_3_ layers enables electrically controlled switching between four regimes, each ensuring excitation of only one mode in the given frequency range. This effect can be used for creating electrically controlled microwave filters that are capable of substantial switching of their operating frequencies.

## Theoryvalidation

The theoretical approach, considered above and in^18–20^, wasverified for devices with fundamentally different operating frequencies (MHz and GHz scales)^15–17^. Theultrasound transducer (operating at MHz frequencies) reported in ref.^16^, could be considered as the first ferroelectric multilayers based device with switchableresonance frequency. Itconsisted of two plates of electrostrictivematerials (solid solution Pb(MgNb)O_3_ and PbTiO_3_) with polarizations controlled independently bythe dc biases of selected polarity. Experimental results for anti-phase and phase vibration mode regimes for layers corresponded approximately resonance frequency switching from f ~ 2.7 MHz to ~4.6 MHz. The calculation basedon the theoretical approach presented above demonstrate similar results: frequency switching from f = 2.6 and 4.7MHz^20^.

**Figure 6:**
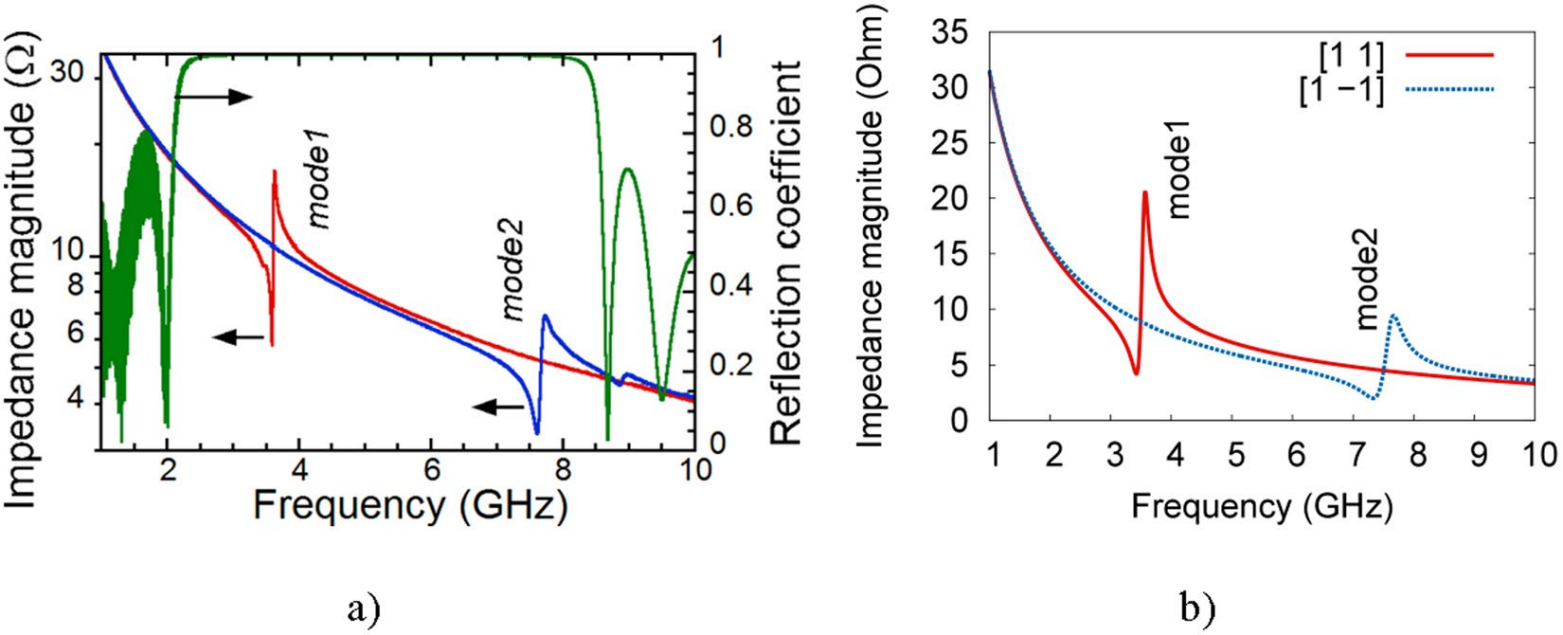
Experimental results^15^ (**a**) and our simulation (**b**) Impedance magnitude of the resonator in the resonant mode1 and mode2. (reproduced from S. Gevorgian and A. Vorobiev, Applied Physics Letters 104, 222905 (2014), with the permission of AIP Publishing).

In ref.^15^, Gevorgian and Vorobiev have reported the firstFBARwith frequency switching ability operating at microwaves. In their work, a composite thin film BAW resonator based on a Ba_0.25_Sr_0.75_TiO_3_/SrRuO_3_/Ba_0.25_Sr_0.75_TiO_3_(180/50/180 nm) multilayer structure was considered. It demonstrates switching of the resonance frequencies by more than two times, from 3.6 GHz (mode1) to 7.7 GHz (mode2), by changing the polarity of the applied bias voltage (5 V dc) at one of the ferroelectric layers (Fig. 6a). Modelling this structure’s response (Fig. 6b), we predicted the resonance and the anti-resonance frequencies with 5% error, which is expectabletaking into account the possible layers’ thickness measurement error.

Finally, the proposed model theoretically explained the improved (5–10 times) quality factor in the frequency range (1–3) GHz of the device, described in patent^17^, as due to the switching of acoustic resonances to the frequency range of ~(4–5) GHz.

## Other possible applications

**Figure 7:**
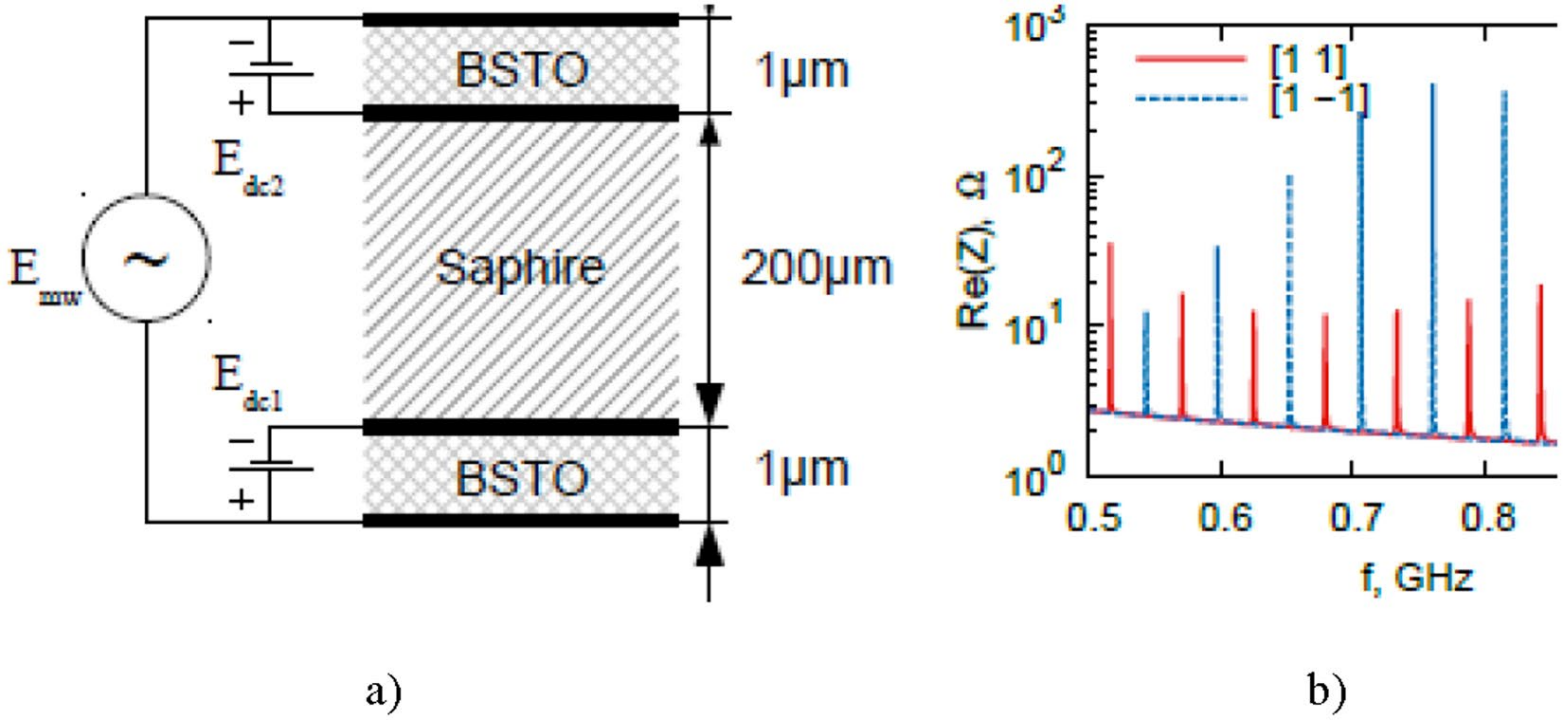
Schematic of high overtone bulk acoustic resonator (**a**), and its frequency spectrum at the same ([1 1]) and opposite ([1–1]) bias polarities on ferroelectric layers (**b**).

The first example is a high overtone bulk acoustic resonator (HBAR) (Fig. 7a) that consists of a substrate (e.g. Al_2_O_3_) coated with BSTO films on both sides. In accordance with our modelling, this could provide switching ability between two sets of normal modes (“odd” and “even”) by changing the polarities of the bias fields applied to the ferroelectric layers (see Fig. 7b). For excitation of odd modes, both ferroelectric layers oscillate in-phase and for even modes –in anti-phase.

**Figure 8:**
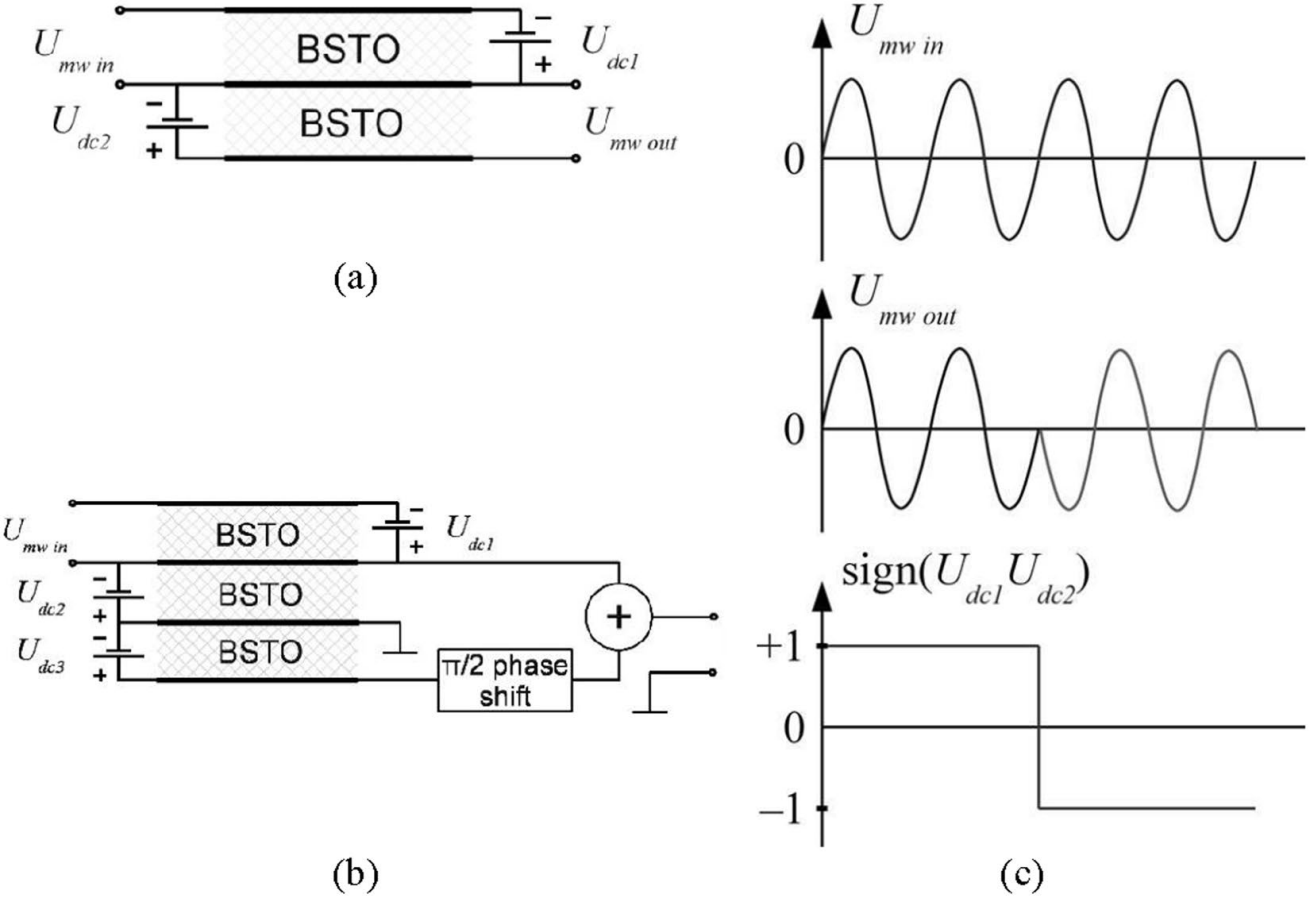
BPSK (**a**) and QPSK (**b**) modulators. Oscillograms illustrate the operation of BPSK modulator (**c**).

Another possible application of the two BSTO layers structure is a simple phase modulator with two phase positions: 0 and π, which allows the BPSK modulation for a set of operating frequencies (Fig. 8(a,c)). With reference to Fig. 8a, the top (input) layer acts as electroacoustic convertor and generates an acoustic wave that propagates through the structure to the bottom (output) layer, which works as acoustoelectric converter and generates the resulting microwave signal. The main mode of this structure is the 2nd, but (like the traditional stacked crystal filters (SCF)^3^) it could also operate on the 1st and 3rd modes. Switching one of the bias polarities applied to the ferroelectric layers leads (due to effect of “negative piezoelectric coefficient”) to switching of the phase of the output signal (for any of these modes) by π degrees. The same principle of operation can be used for development of a three FE layers QPSK modulator (Fig. 8b).

## Conclusion

The theory of selective excitation of acoustical eigenmodes in multilayer ferroelectric film structures allows us to design a new class of microwave film bulk acoustic resonators (FBAR) with the possibility of substantial frequency switching. Also, a so-called “criterion function” is suggested that allows to determine the conditions for effective excitations at one selected resonance mode with suppression of the other modes. Finally, in addition to the possible application of the switchable MW FBAR as filter circuits and MW phase modulators, the principle of E-field induced piezo-effect frequency switching can be further expanded and used for development of novel class of devices e.g. voltage control oscillators (VCO), surface acoustic wave (SAW) delay lines, acoustic sensors, acousto-optic devices and impedance transformers.
